# Development of a deep learning‐based nomogram for predicting lymph node metastasis in cervical cancer: A multicenter study

**DOI:** 10.1002/ctm2.938

**Published:** 2022-07-15

**Authors:** Yujia Liu, Hui Duan, Di Dong, Jiaming Chen, Lianzhen Zhong, Liwen Zhang, Runnan Cao, Huijian Fan, Zhumei Cui, Ping Liu, Shan Kang, Xuemei Zhan, Shaoguang Wang, Xun Zhao, Chunlin Chen, Jie Tian

**Affiliations:** ^1^ School of Artificial Intelligence University of Chinese Academy of Sciences Beijing China; ^2^ CAS Key Laboratory of Molecular Imaging, the State Key Laboratory of Management and Control for Complex Systems, Institute of Automation Chinese Academy of Sciences Beijing China; ^3^ Department of Obstetrics and Gynecology, Nanfang Hospital Southern Medical University Guangzhou China; ^4^ Beijing Key Laboratory of Molecular Imaging Beijing China; ^5^ Huizhou Municipal central Hospital Huizhou China; ^6^ The affiliated hospital of Qingdao University Qingdao China; ^7^ Department of Gynecology Fourth Hospital Hebei Medical University Shijiazhuang China; ^8^ Jiangmen central Hospital Jiangmen China; ^9^ Department of Gynecology Yantai Yuhuangding Hospital Yantai China; ^10^ Beijing Advanced Innovation Center for Big Data‐Based Precision Medicine, School of Engineering Medicine Beihang University Beijing China; ^11^ Zhuhai Precision Medical Center Zhuhai People's Hospital (Affiliated with Jinan University) Zhuhai China

Dear Editor,

Cervical cancer is one of the most frequently diagnosed cancers in women and has a high mortality rate worldwide.^1^ Lymph node metastasis (LNM) is an important prognostic factor in patients with cervical cancer.^2^, ^3^, ^4^ The assessment of LNM before treatment is essential to guide and tailor the treatment.^5^, ^6^ The morphological examination of lymph nodes via medical images is commonly used for diagnosing LNM. However, it depends mainly on radiologists’ experience and has relatively low accuracy. Thus, we collected a multi‐center dataset and developed a deep learning‐based nomogram (DLN) to improve the accuracy of LNM diagnosis in cervical cancer.

In total, 1123 cervical cancer patients with computed tomography (CT) examination were enrolled from 13 centers in our study (Table S1 and Supplementary A1). As shown in Supplementary A2 and Figure S1, we divided these patients into four cohorts: training cohort, validation cohort, external testing cohort 1, and external testing cohort 2. Detailed information on the four cohorts is presented in Table S2. The clinical characteristics included age, gravidity, histological type, FIGO stage, etc. Moreover, two experienced gynecologists, who were blinded to the pathological report, were invited to diagnose the status of LNM together using only CT images. Additionally, a follow‐up cohort including 148 patients from one center was used for survival analysis.

The workflow of this study is described in Figure 1, including region of interest (ROI) segmentation, data preprocessing (Supplementary A3), model construction, and model evaluation (Supplementary A4).

**FIGURE 1:**
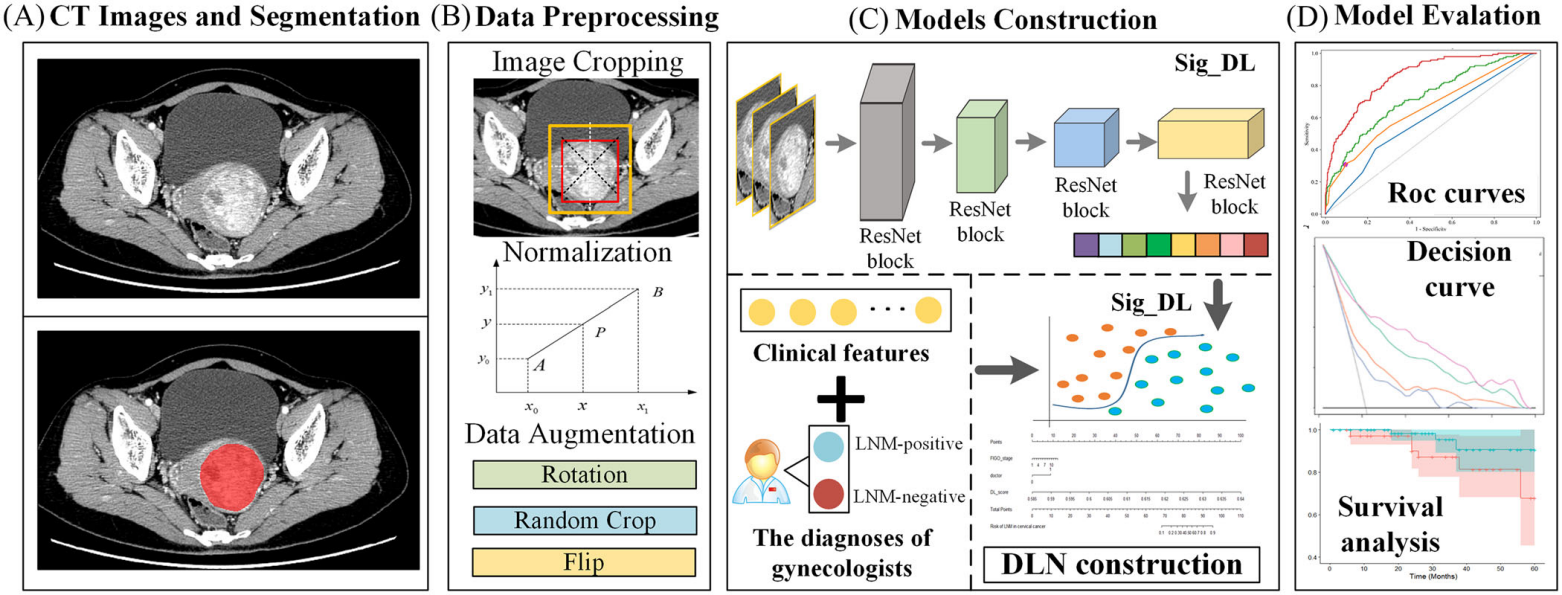
Workflow of the development of deep learning‐based nomogram (DLN). (A) CT images and segmentation, (B) Data preprocessing. (C) Model construction. (D) Model evaluation

We invited experienced gynecologists to segment ROIs in normalized CT images. Before model construction, data augmentations, including flipping, rotating, and random cropping, were used to generate new training samples to avoid overfitting. Oversampling methods were used to balance the ratio of LNM‐positive patients and LNM‐negative patients in the training cohort. Three state‐of‐the‐art deep learning methods, including ResNet18,^7^ ResNet50,^7^ and SE‐Net,^8^ were used to construct three candidate models (Supplementary A5). As shown in Table S3, ResNet18 showed the best performance in the validation cohort, and thus it was selected to build the final deep learning signature (Sig_DL). As shown in Supplementary A6, a total of 1407 handcrafted radiomic features were extracted, and three key radiomic features were selected via a series of feature selection methods and integrated them into a radiomic signature (Sig_radiomic).^9^, ^10^ As shown in Table 1 and Figure S2, the AUCs of Sig_DL performed better than Sig_radiomic in all the cohorts.

**TABLE 1:** Performance of models in all cohorts

	Specificity	Sensitivity	Accuracy	AUC (95% CI)	True negative	True positive	False negative	False positive
Sig_clin								
Training cohort	0.578	0.678	0.600	0.678 (0.619–0.726)	289	97	46	218
validation cohort	0.710	0.423	0.665	0.597 (0.481–0.722)	97	11	15	41
External testing1	0.579	0.348	0.544	0.489 (0.367–0.605)	73	8	15	53
External testing2	0.615	0.572	0.609	0.626 (0.485–0.755)	80	12	9	50
Sig_radiomic								
Training cohort	0.527	0.545	0.531	0.575 (0.520–0.626)	269	78	65	247
validation cohort	0.522	0.577	0.530	0.621 (0.505–0.746)	72	15	11	66
External testing 1	0.508	0.652	0.530	0.616 (0.497–0.735)	64	15	8	62
External testing 2	0.562	0.619	0.570	0.595(0.475–0.714)	73	13	8	57
Sig_DL								
Training cohort	0.734	0.818	0.753	0.853 (0.821–0.885)	379	117	26	137
validation cohort	0.710	0.731	0.713	0.787 (0.702–0.878)	98	19	7	40
External testing 1	0.651	0.739	0.664	0.776 (0.677–0.877)	82	17	6	29
External testing 2	0.777	0.714	0.768	0.768 (0.662–0.874)	101	15	6	29
DLN^a^								
Training cohort	0.793	0.790	0.792	0.867 (0.839–0.897)	412	113	30	104
validation cohort	0.783	0.654	0.762	0.807 (0.713–0.889)	108	16	10	30
External testing 1	0.714	0.739	0.718	0.781 (0.669–0.876)	91	17	6	35
External testing 2	0.808	0.667	0.788	0.804 (0.705–0.892)	105	14	7	25

^a^ DLN, deep learning‐based nomogram.

Additionally, univariate analysis was used to screen for significant clinical features. We noticed that the FIGO stage was significantly associated with LNM (P < 0.01). After multivariable logistic regression, we selected the FIGO stage and age as key clinical features and used them to construct a clinical signature (Sig_clin). The area under the receiver operating characteristic curve (AUCs) of Sig_clin reached 0.678 and 0.597 in training and validation cohorts, respectively.

Finally, we integrated Sig_DL, diagnoses of gynecologists, and all significant clinical features into a DLN via multivariate linear regress analysis (Table S4 and Figure 2A). Compared with other models, DLN had the best predictive ability (Figure S3), with AUCs of 0.867, 0.807, 0.781, and 0.804 in the training cohort, validation cohort, external testing cohort1 and external testing cohort2 (Figure 2B–E). As shown in Table 1, the accuracy also indicated the good performance of DLN in these four cohorts.

**FIGURE 2:**
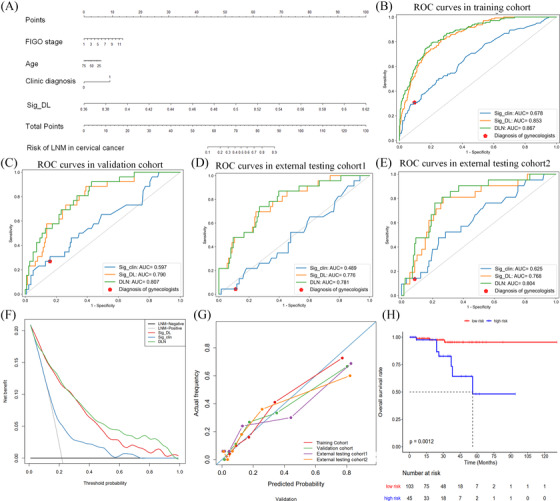
Evalutaion of the deep learning‐based nomogram (DLN) mdoel. (A) The DLN model. The ROC curves in the training cohort (B), validation cohort (C), external testing cohort 1(D), and external testing cohort 2 (E). (F) The decision curve of all models. (G) The calibration curves of the DLN. (H) Kaplan–Meier curves of DLN in the follow‐up cohort. ROC, receiver operating characteristic curve

Meanwhile, the decision curves showed that the patients could benefit more from DLN than both Sig_DL and Sig_clin (Figure 2F). As shown in Figure 2G, the calibration curves demonstrated that the DLN had good consistency with the gold standard of LNM.

It is worth noting that the diagnoses of the gynecologists had high specificity but low sensitivity in our cohorts. Therefore, we modified the cutoff value so that DLN could have the same specificity as the gynecologists’ diagnoses. Then, we found that DLN had better accuracy and sensitivity than the gynecologists (Table S5). The Venn diagrams also showed that DLN had more true positive cases than the gynecologists (Figure S4). Four typical cases are shown in Figure 3, which indicates that DLN could help the clinician reduce the risk of misdiagnosis.

**FIGURE 3:**
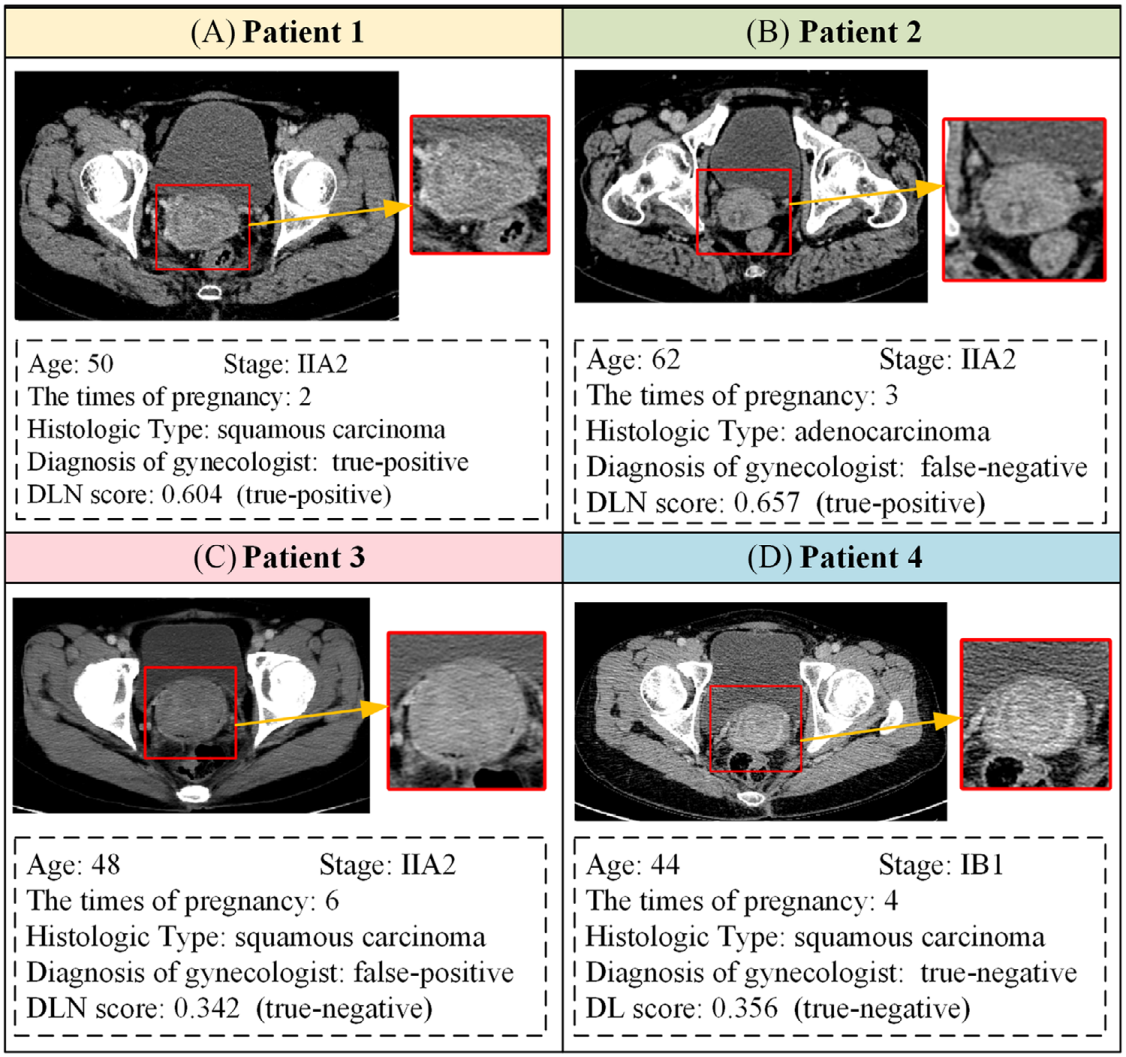
Four typical cases to show the performance of deep learning‐based nomogram (DLN) and gynecologists. (A) A lymph node metastasis (LNM)‐positive patient correctly diagnosed by both DLN and gynecologists; (B) An LNM‐positive patient only correctly diagnosed by DLN; (C) An LNM‐negative patient only correctly diagnosed by DLN; (D) An LNM‐positive patient correctly diagnosed by both DLN and gynecologists

Subgroup analysis was performed on the data of the enrolled patients, including their clinical characteristics, the CT manufacturers, and the centers. As shown in Figure S5A–F, the subgroup analysis indicates that the DLN was not affected by age, times of pregnancy, human papillomavirus (HPV) testing result, and histological type. Especially, we selected 614 cervical cancer patients for human papillomavirus (HPV) testing. Subgroup analysis revealed that our DLN showed good performance in both HPV‐positive subgroup and HPV‐negative subgroup (Figure S5G–H). Our model also was minimally affected by the CT manufacturers and centers (Figure S6A,B).

Besides, 148 cervical cancer patients with follow‐up from Center 2 were used for exploring the association between DLN score and overall survival (OS) using Kaplan‐Meier curves (Supplementary A7). We divided them into low‐risk and high‐risk groups using the mean value of DLN score as a cutoff. As shown in Figure 2H, we found that the high‐risk group exhibited shorter OS (log‐rank test: *P* = 0.0012). Furthermore, we stratified patients via the FIGO stage for comparison, however, the FIGO stage showed no significant association with OS (Figure S7). Hence, DLN could serve as a significant prognostic factor for cervical cancer.

In conclusion, we developed a deep learning model for the preoperative prediction of LNM in cervical cancer and validated it in a large‐scale and multicenter dataset. The performance of DLN surpassed the diagnosis of experienced gynecologists. Therefore, DLN can serve as a non‐invasive tool for LNM determination and thus assist treatment decision‐making.

## CONFLICT OF INTEREST

The authors declare no conflict of interest.

## Supporting information

Supplementary A1. The inclusion and exclusion criteria of this study
Supplementary A2. The dataset partition and sample size estimation
Supplementary A3. Region of interest segmentation and data preprocessing
Supplementary A4. Evaluation of the models
Supplementary A5. Training details of the three deep learning networks
Supplementary A6. Handcrafted features extraction and Sig_radiomic building
Supplementary A7. The prognostic analysis of DLN
Table S1. Detailed information of the data in each center
Table S2. Clinical characteristics in the training cohort, validation cohort and external testing cohorts
Table S3. Performance of deep learning and radiomic signatures in all cohorts
Table S4. The logistic linear regression of features in DLN
Table S5. Performance of the DLN and the diagnoses of gynecologists in all cohorts.
Figure S1. The Flowchart of this multicenter study.
Figure S2. The ROC curves of different signatures in all cohorts.
Figure S3. The performance of the constructed models in all cohorts.
Figure S4. Venn diagram comparing the performance of DLN with the diagnoses of gynecologists.
Figure S5. Subgroup analysis of clinical characteristics.
Figure S6. Subgroup analysis on (A)different centers and (B) different CT manufacturers.
Figure S7. Kaplan‐Meier curve of overall survival for FIGO stage in follow‐up cohort.
