# Supplementary material for: Development of a deep learning‐based nomogram for predicting lymph node metastasis in cervical cancer: A multicenter study
Source: Clin Transl Med. 2022 Jul 15;12(7):e938. doi: 10.1002/ctm2.938 (PMC9286523; doi:10.1002/ctm2.938)

**Supplementary A1. The inclusion and exclusion criteria of this study**

In total, 1123 cervical cancer patients were enrolled from 13 centers in our study. The inclusion criteria of this study were as follows: a) pathologically confirmed cervical carcinoma; b) LNM statuses confirmed by postoperative pathology; c) no history of preoperative adjuvant therapy; d) preoperative enhanced venous phase CT images and clinical data are available. The exclusion criteria included: a) CT scans more than 2 weeks before surgery; b) with other malignant tumors.

**Supplementary A2. The dataset partition and sample size estimation**

In this multicenter study, we collected 1123 patients from 13 different centers (Table S1). We divided all the patients into four study cohorts: training cohort, validation cohort, and two external testing cohorts. The detailed data division criteria are as follows:

We selected 7 centers, whose patient number is more than 100 or less than 20 patients, and randomly divided these patients into training and validation cohorts in a ratio of 4:1. Then, the remaining centers’ patients were included in the two external testing cohorts.

Note that the training cohort was used to train the parameters in the model, the validation cohort was performed to optimize the model’s parameters, and the external testing cohorts were carried out to evaluate the performance and generalization ability of the model.

In order to accurately evaluate the performance of the model, the minimum size of testing samples in the external testing cohort was calculated [1]. We used the following formula to test whether the means of two groups are significantly different.

Assume that the mean of two groups (group A and B) satisfies the following conditions:

$$H_{0}: \mu_{A}-\mu_{B}=0$$

$$H_{1}: \mu_{A}-\mu_{B}\neq0$$

$\mu_{A}$ and $\mu_{B}$ represent the mean value in group A and group B, respectively.

$$N_{A}=\left( \frac{n_{A}+n_{B}}{n_{B}} \right)\left( \sigma\frac{z_{1-\alpha/2}+z_{1-\beta}}{\mu_{A}-\mu_{B}} \right)^{2}$$

$$N_{B}=\left( \frac{n_{A}+n_{B}}{n_{A}} \right)\left( \sigma\frac{z_{1-\alpha/2}+z_{1-\beta}}{\mu_{A}-\mu_{B}} \right)^{2}$$

$1-\beta=\Phi\left( z-z_{1-\alpha/2} \right)+\Phi\left( -z-z_{1-\alpha/2} \right)$, $z=\frac{\mu_{A}-\mu_{B}}{\sigma\sqrt{\frac{1}{n_{A}}+\frac{1}{n_{B}}}}$

Note, $n$ means the sample size in the training cohort and $N$ is the sample size for the testing cohort, $\Phi$ is the standard Normal distribution function. $\alpha$ is the Type I error; we set it to 0.005; $\beta$ is the Type II error, we set it to 0.05. $1-\beta$ is the power, and $\sigma^{2}$ is the variance of the covariate.

In this study, we found the sample size needed in the external testing cohort was 21 patients with LNM-positive and 60 patients with LNM-negative. Considering the patient number in each center, we combined the patients from centers 8-9 into the external testing cohort 1 and patients from centers 10-13 into the external testing cohort 2.

**Supplementary A3. Region of interest segmentation and data** **preprocessing**

The region of interest (ROI) was defined as the tumor area. Some studies have shown that the performance of models constructed by 2D ROI is similar to that built by 3D ROI. In view of this, we chose 2D ROIs for analysis [1][2]. A 5-year experienced gynecologist selected the slice with the largest tumor area from CT images and segmented tumor ROI using ITK-SNAP software (<http://www.itksnap.org/>). Additionally, all the ROIs were checked by another gynecologist with 10 years of experience. We cropped a rectangular box with a size of 100ⅹ100, covering the ROI for subsequent image analysis. CT images from all centers were interpolated linearly to the pixel size of 1mm×1mm×1mm and then normalized using the z-score method [3].

**Supplementary A4. Evaluation of the models**

All models were constructed in the training cohort, optimized in the validation cohort, and validated in the two external validation cohorts. The area under the receiver operating characteristic (ROC) curve (AUC) was used as the main evaluation metric to measure the prediction ability of the models. Besides, we also selected the cutoff value when the Youden index achieved the maximum value and calculated the specificity, sensitivity, accuracy, true positivity (TP), and true negativity (TN) of the models. Decision curve analysis was performed for all models. A Venn diagram was applied to visually evaluate and compare the performance of DLN with the diagnoses of the gynecologists.

All the models were built by Python (version 3.7.6, <https://www.python.org/>). In the univariate analysis, we used the Mann-Whitney U test to analyze continuous variables and the Wilcoxon signed-rank test for categorical factors. P < 0.05 was regarded to show statistical significance. All the statistical analyses were performed by the R software (R version 3.6.0, <https://www.r-project.org/>).

**Supplementary A5. Training details of the three deep learning networks**

CT images from all centers were interpolated linearly and then normalized using the z-score method[3]. Data augmentations, including flipping, rotating, and random cropping, were used to generate new training samples for avoiding overfitting[4]. During the training, we augmented the data via 1) randomly flipping the images vertically or horizontally with a probability of 0.5; 2) randomly rotating with a probability of 0.5; 3) randomly scaling the volumes to 0.9-1.1 and then randomly cropping them to the size of 100 × 100. Finally, we resized the data into 224 × 224 as the model inputs. In addition, we used oversampling methods to balance the ratio of LNM-positive patients and LNM-negative patients in the training cohort.

In order to acquire the best performance of the deep learning model, we constructed three models using different deep learning networks (including ResNet18, ResNet50, and SE-Net). All three networks were first pre-trained by the ImageNet dataset. Then, the resized ROIs were sent into pre-trained networks for automatically extracting deep learning features and modeling. During the model training, the ROI images in the validation cohort were used to optimize the hyper-parameters to achieve the best prediction ability.

We used a batch size of 64 and the binary cross-entropy loss function. The Adam optimizer was used to update the weights of the model. The learning rate of the first 20 epochs was 1e-4, which was then changed to 1e-5 after 20 epochs. Model training was stopped when a number of epochs did not show improvement.

Note that, the models we built in this study are available on our website (www.radiomics.net.cn/platform.html).

**Supplementary A6. Handcrafted features extraction and Sig_radiomic building**

We extracted radiomic features according to the guidance of the Image Biomarker Standardization Initiative (IBSI) [6]. By the way, all feature extraction methods were implemented using Pyradiomics (https://pyradiomics.readthedocs.io/en/latest/). A total of 1407 radiomic features were extracted from ROIs, including 12 shape features, 75 neighboring gray tone difference matrix (NGTDM) features, 210 gray-level co-occurrence matrix (GLDM) features, 240 Gy-level size zone matrix (GLSZM) features, 240 Gy-level run length matrix (GLRLM) features, 270 first-order features, and 270 gray-level co-occurrence matrix (GLCM) features.

Radiomic features were normalized using the z-score method for each cohort. To reduce the feature dimension and prevent the model from overfitting, the radiomic features having significant associations with LNM were first selected using univariable analysis. Then, minimum redundancy maximum relevance (mRMR) [7] methods were applied to rank and select key features. Finally, the selected key features were fed into the multivariable logistic regression model to construct the radiomic signature (Sig_radiomic).

The three key features in Sig_radiomic included gradient_ngtdm_Contrast (NGTDM feature), gradient_glszm_SizeZoneNonUniformityNormalized (GLSZM feature), and square_glrlm_RunLengthNonUniformity (GLRLM feature). The performance of Sig_radiomic is shown in Supplementary Table S2.

**Supplementary A7. The prognostic analysis of DLN**

To investigate the correlation between the status of LNs and prognosis, survival analysis was carried out to evaluate the prognosis of our proposed model. We first stratified patients into high-risk group and low-risk group using the mean value of DLN score as cutoff. The DLN score was significantly associated with OS (hazard ratio, 5.27; 95%CI, 0.1.395-19.93; P =0.0012). Patients with higher DLN scores had a shorter OS time.

Moreover, we also test the prognostic value of FIGO stage. We divided patients into two groups according to the FIGO stage: a high-risk group (FIGO stage I), and a low-risk group (FIGO stage II). As shown in Figure S7, we found that there was no significant difference of OS between FIGO stage I group and FIGO stage II group (hazard ratio, 1.546; 95% CI, 0.189-2.214; P =0.49).

However, although this study exploited survival analysis to further reveal the prognostic value for our model, the sample size was limited. In future studies, we will collect larger follow-up data to further explore the prognostic value of our model.

**Reference**

1. Chow S C, Shao J, Wang H, et al. Sample size calculations in clinical research. chapman and hall/CRC, 2017.
2. Meng L, Dong D, Chen X, et al. 2D and 3D CT radiomic features performance comparison in characterization of gastric Cancer: a multi-center study. IEEE journal of biomedical and health informatics, 2020, 25(3): 755-763.
3. Arefan D, Chai R, Sun M, et al. Machine learning prediction of axillary lymph node metastasis in breast cancer: 2D versus 3D radiomic features. Medical physics, 2020, 47(12): 6334-6342.
4. Cheng S, Fang M, Cui C, et al. LGE-CMR-derived texture features reflect poor prognosis in hypertrophic cardiomyopathy patients with systolic dysfunction: preliminary results. European radiology, 2018, 28(11): 4615-4624.
5. Zhong L Z, Fang X L, Dong D, et al. A deep learning MR-based radiomic nomogram may predict survival for nasopharyngeal carcinoma patients with stage T3N1M0. Radiotherapy and Oncology 2020; 151: 1-9.
6. wanenburg A, Leger S, Vallières M, et al. Image biomarker standardisation initiative[J]. arXiv preprint arXiv:1612.07003, 2016.
7. Peng H, Long F, Ding C. Feature selection based on mutual information criteria of max-dependency, max-relevance, and min-redundancy[J]. IEEE Transactions on pattern analysis and machine intelligence, 2005, 27(8): 1226-1238.

**Table S1. Detailed information of the data in each center**

| Center names | Index | Number of patients | LNM-positive | LNM-negative | Cohorts |
| --- | --- | --- | --- | --- | --- |
| The Affiliated Hospital of Qingdao University | Center 1 | 255 | 50 | 205 | Train/validation cohort |
| Nanfang Hospital | Center 2 | 219 | 39 | 180 | Train/validation cohort |
| Jiangmen Central Hospital | Center 3 | 197 | 17 | 180 | Train/ validation cohort |
| The fourth hospital of Hebei Medical University | Center 4 | 129 | 60 | 69 | Train/ validation cohort |
| Gansu Provincial Hospital | Center 5 | 9 | 1 | 7 | Train/ validation cohort |
| The Second Affiliated Hospital of Zhengzhou University | Center 6 | 9 | 1 | 8 | Train/ validation cohort |
| Yuncheng Central Hospital | Center 7 | 6 | 1 | 5 | Train/ validation cohort |
| Yuhuangding hospital | Center 8 | 76 | 17 | 59 | External testing cohort 1 |
| Guizhou Provincial Hospital | Center 9 | 73 | 6 | 67 | External testing cohort 1 |
| Ningbo Maternal and Child Health Care Hospital | Center 10 | 44 | 3 | 41 | External testing cohort 2 |
| The Third Affiliated Hospital of the Third Military Medical University | Center 11 | 43 | 5 | 37 | External testing cohort 2 |
| The First Affiliated Hospital of Zhengzhou University | Center 12 | 43 | 7 | 36 | External testing cohort 2 |
| Nanchong Central Hospital | Center 13 | 22 | 6 | 16 | External testing cohort 2 |

**Table S2.** **Clinical characteristics in the training cohort, validation cohort and external testing cohorts**

|  | Training cohort (n= 659) | | | Validation cohort (n=164) | | | External testing 1 (n=149) | | | External testing 2 (n=151) | | |
| --- | --- | --- | --- | --- | --- | --- | --- | --- | --- | --- | --- | --- |
|  | LNM^a^- positive  (n=143) | LNM- negative  (n=516) | *P* | LNM-positive  (n=26) | LNM-negative  (n=138) | *P* | LNM-positive  (n=23) | LNM-negative  (n=126) | *P* | LNM-positive  (n=21) | LNM-negative  (n=130) | *P* |
| Age, mean (range) | 48.46  (28-75) | 48.91  (24-71) | 0.33 | 44.88  (32-60) | 48.37  (26-68) | 0.09 | 47.04  (34-67) | 46.99  (25-65) | 0.91 | 43.32  (27-56) | 45.32  (22-66) | 0.64 |
| Pregnancy,  mean (range) | 3.54  (1-14) | 3.29  (0-10) | 0.21 | 3.00  (0-7) | 3.39  (1-10) | 0.38 | 2.83  (0-5) | 3.2-9  (1-8) | 0.32 | 2.84  (1-6) | 3.31  (1-10) | 0.13 |
| FIGO stage |  |  | <0.01 |  |  | 0.19 |  |  | 0.47 |  |  | 0.06 |
| IA | 0 |  |  | 0 | 1 |  | 0 | 1 |  | 0 | 3 |  |
| IA1 | 0 | 3 |  | 0 | 0 |  | 0 | 2 |  | 0 | 0 |  |
| IA2 | 1 | 12 |  | 0 | 5 |  | 0 | 3 |  | 0 | 4 |  |
| IB1 | 84 | 379 |  | 17 | 104 |  | 13 | 71 |  | 10 | 80 |  |
| IB2 | 21 | 32 |  | 5 | 7 |  | 1 | 11 |  | 1 | 13 |  |
| IIA1 | 27 | 69 |  | 2 | 11 |  | 8 | 31 |  | 9 | 24 |  |
| IIA2 | 9 | 19 |  | 2 | 9 |  | 1 | 7 |  | 1 | 5 |  |
| IIB | 1 | 2 |  | 0 | 1 |  | 0 | 0 |  |  | 1 |  |
| Histological type |  |  | 0.67 |  |  | <0.01 |  |  | 0.43 |  |  | 1 |
| Adenocarcinoma | 20 | 68 |  | 5 | 37 |  | 4 | 18 |  | 2 | 13 |  |
| Adenosquamous | 4 | 8 |  | 4 | 1 |  | 1 | 1 |  |  | 2 |  |
| Squamous carcinoma | 118 | 432 |  | 17 | 98 |  | 18 | 104 |  | 19 | 114 |  |
| Other types | 1 | 8 |  |  | 2 |  | 0 | 3 |  | 0 | 1 |  |
| HPV^a^ |  |  |  |  |  |  |  |  |  |  |  |  |
| Positive | 44 | 252 |  | 11 | 66 |  | 8 | 64 |  | 7 | 39 |  |
| Negative | 16 | 66 |  | 3 | 13 |  | 2 | 5 |  | 2 | 15 |  |
| Unreported | 83 | 198 |  | 12 | 59 |  | 13 | 57 |  | 12 | 76 |  |

^ab^LNM=lymph node metastasis.

^b^HPV= human papilloma virus.

**Table S3. Performance of deep learning and radiomic signatures in all cohorts**

| Index | Specificity | Sensitivity | Accuracy | AUC (95%CI) | TN | TP | FN | FP |
| --- | --- | --- | --- | --- | --- | --- | --- | --- |
| ResNet18 (Sig_DL) |  |  |  |  |  |  |  |  |
| Training cohort | 0.734 | 0.818 | 0.753 | 0.853 (0.821-0.885) | 379 | 117 | 26 | 137 |
| validation cohort | 0.710 | 0.731 | 0.713 | 0.787 (0.702-0.878) | 98 | 19 | 7 | 40 |
| External testing 1 | 0.651 | 0.739 | 0.664 | 0.776 (0.677-0.877) | 82 | 17 | 6 | 29 |
| External testing 2 | 0.777 | 0.714 | 0.768 | 0.768 (0.662-0.874) | 101 | 15 | 6 | 29 |
| ResNet50 |  |  |  |  |  |  |  |  |
| Training cohort | 0.616 | 0.664 | 0.627 | 0.672 (0.625-0.723) | 328 | 90 | 53 | 188 |
| validation cohort | 0.645 | 0.769 | 0.665 | 0.734 (0.613-0.813) | 89 | 20 | 6 | 49 |
| External testing 1 | 0.611 | 0.609 | 0.611 | 0.620 (0.488-0.753) | 77 | 14 | 9 | 49 |
| External testing 2 | 0.562 | 0.667 | 0.576 | 0.585 (0.457-0.714) | 73 | 14 | 7 | 57 |
| SeNet18 |  |  |  |  |  |  |  |  |
| Training cohort | 0.690 | 0.762 | 0.706 | 0.771 (0.728-0.811) | 356 | 109 | 34 | 160 |
| validation cohort | 0.630 | 0.654 | 0.634 | 0.773 (0.666-0.869) | 87 | 17 | 9 | 51 |
| External testing 1 | 0.722 | 0.696 | 0.718 | 0.727 (0.608-0.847) | 91 | 16 | 7 | 35 |
| External testing 2 | 0.746 | 0.476 | 0.709 | 0.688 (0.570-0.806) | 97 | 10 | 11 | 33 |
| Sig_radiomic |  |  |  |  |  |  |  |  |
| Training cohort | 0.527 | 0.545 | 0.531 | 0.575 (0.520-0.626) | 269 | 78 | 65 | 247 |
| validation cohort | 0.522 | 0.577 | 0.530 | 0.621 (0.505-0.746) | 72 | 15 | 11 | 66 |
| External testing 1 | 0.508 | 0.652 | 0.530 | 0.616 (0.497-0.735) | 64 | 15 | 8 | 62 |
| External testing 2 | 0.562 | 0.619 | 0.570 | 0.595(0.475-0.714) | 73 | 13 | 8 | 57 |

**Table S4. The logistic linear regression of features in DLN**

| Features | *P* value | Coefficient |
| --- | --- | --- |
| Age | 0.2318 | -0.01535 |
| FIGO stage | 0.00353 | 0.1779 |
| Diagnosis of gynecologist | 0.0000137 | 1.311 |
| Sig_DL | <1e-6 | 54.99 |
| Intercept | <1e-6 | -29.18 |

**Table S5. Performance of the DLN and the diagnoses of gynecologists in all cohorts. We keep the specificities of DLN and the diagnoses of gynecologists the same.**

|  | Specificity | Sensitivity | Accuracy | TN^a^ | TP^b^ | FN^c^ | FP^d^ |
| --- | --- | --- | --- | --- | --- | --- | --- |
| Diagnoses of gynecologist |  |  |  |  |  |  |  |
| Training cohort | 0.905 | 0.308 | 0.776 | 468 | 44 | 99 | 49 |
| Validation cohort | 0.841 | 0.269 | 0.75 | 116 | 7 | 19 | 22 |
| External testing cohort 1 | 0.889 | 0.043 | 0.758 | 112 | 1 | 22 | 14 |
| External testing cohort 2 | 0.915 | 0.136 | 0.803 | 119 | 3 | 19 | 11 |
| DLN |  |  |  |  |  |  |  |
| Training cohort | 0.907 | 0.517 | 0.822 | 468 | 74 | 69 | 48 |
| validation cohort | 0.841 | 0.577 | 0.799 | 116 | 15 | 11 | 22 |
| External testing cohort 1 | 0.889 | 0.348 | 0.805 | 112 | 8 | 15 | 14 |
| External testing cohort 2 | 0.915 | 0.238 | 0.821 | 119 | 5 | 16 | 11 |

^a^TN=true negative.

^b^TP=true positive.

^c^FN=false negative.

^d^FP=false positive.

**Figure S1. The Flowchart of this multicenter study.**


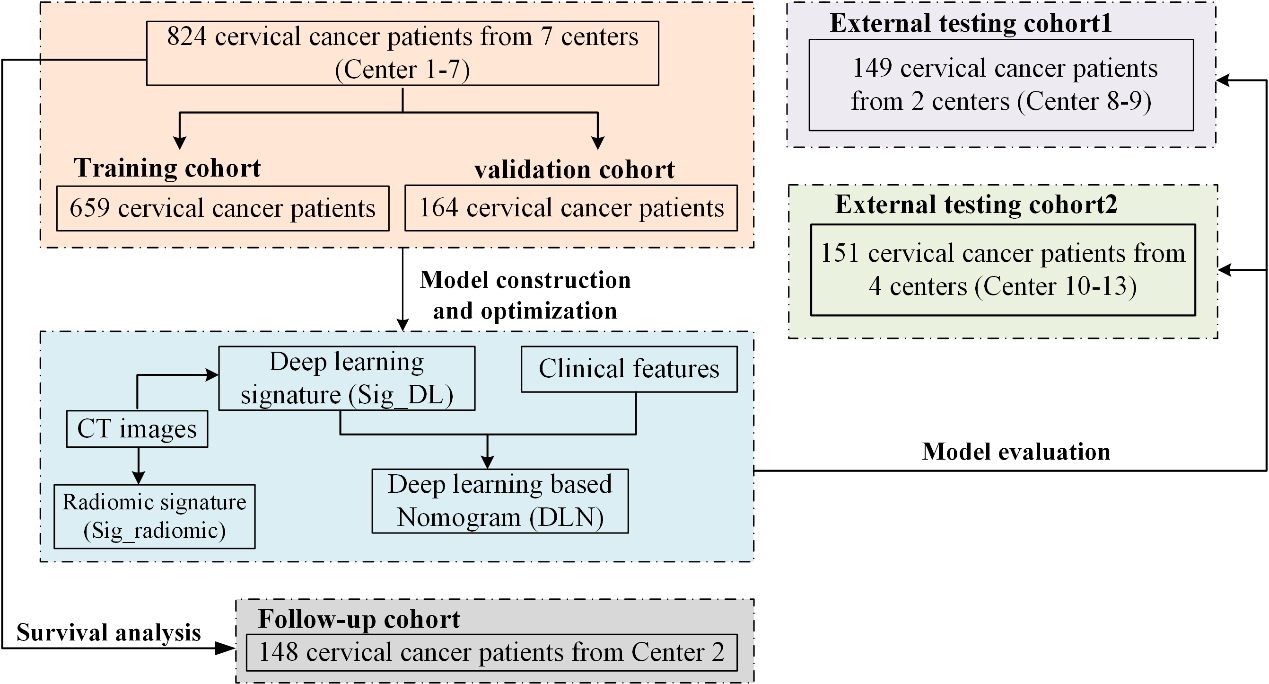


**Figure S2. The ROC curves of different signatures in all cohorts. The ROC curves of Sig_DL, Sig_radiomic, and other state-of-art methods in the training cohort (A), validation cohort (B), external testing cohort 1 (C), and external testing cohort 2 (D).**


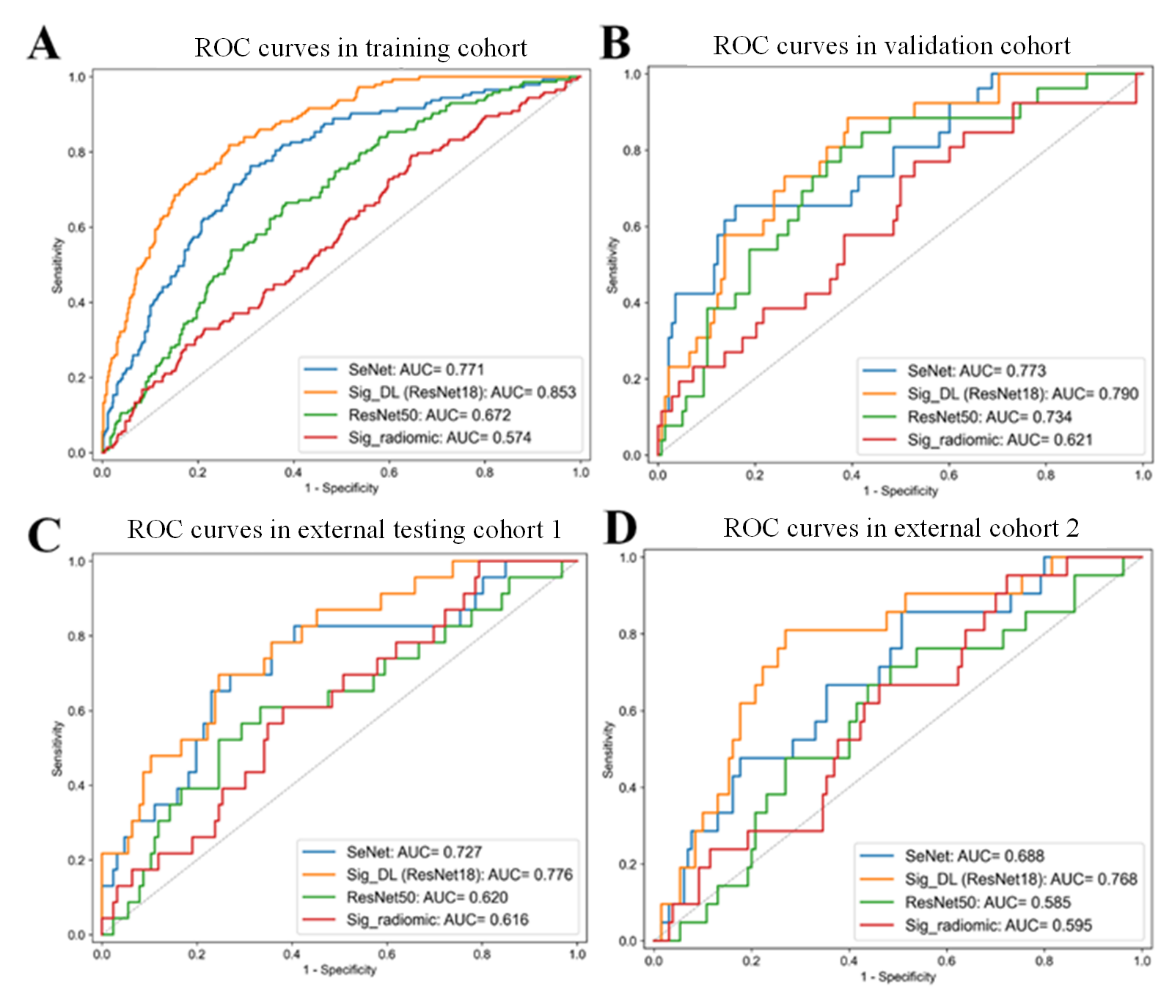


**Figure S3. The performance of the constructed models in all cohorts.**


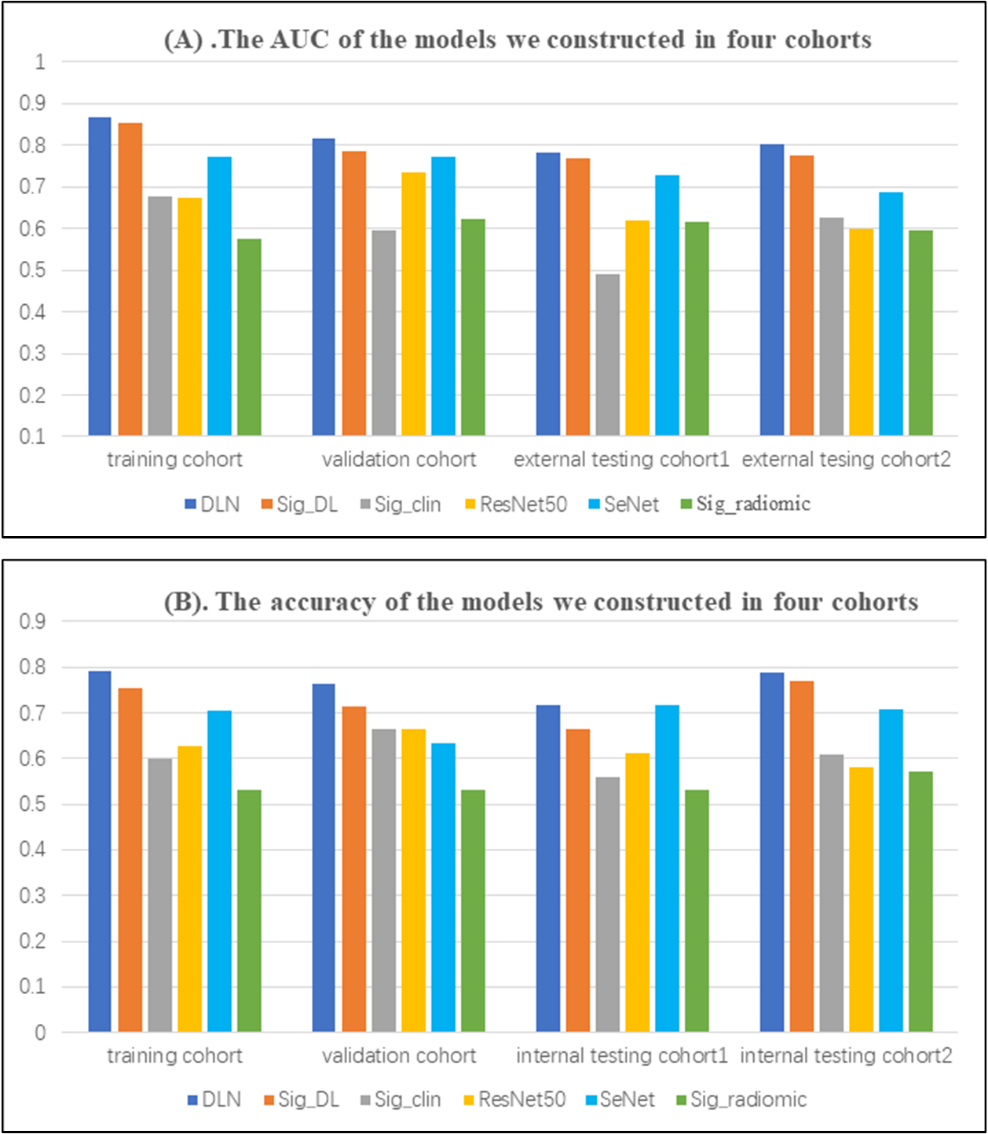


**Figure S4. Venn diagram comparing the performance of DLN with the diagnoses of gynecologists. True negative (TN) cases in training cohort (A), validation cohort (B), external testing cohort 1 (C), and external testing cohort 2 (D). True positive (TP) cases in training cohort (E), validation cohort (F), external testing cohort 1 (G), and external testing cohort 2 (H). The green part represents the number of TPs (or TNs) found by the DLN alone. The red part represents the diagnoses by the gynecologists alone. Overlapping region (orange part) represents the number of TPs (or TNs) found by both the gynecologists and DLN.**


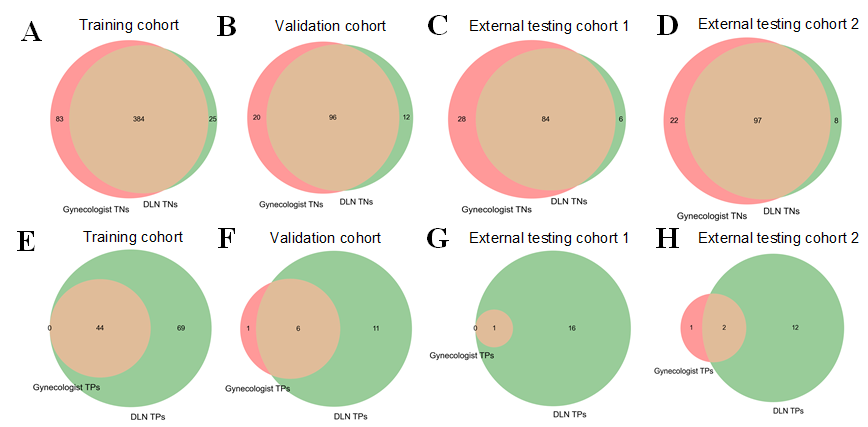


TPs, true positives; TNs, true negatives

**Figure S5. Subgroup analysis of clinical characteristics. (A) The ROC curves of DLN in subgroups with different ages. (B) The distribution of LNM-positive and LNM-negative patients at different ages. (C) The ROC curves of DLN in subgroups with different pregnancy times. (D) The distribution of LNM-positive and LNM-negative patients at different pregnancy times. (E) The ROC curves of DLN in subgroups with different histological types. (F) The distribution of DLN score in different histological types. (G) The ROC curves of DLN in subgroups with different HPV testing results. (H) The distribution of LNM-positive and LNM-negative patients in different HPV testing results. Class 0 means LNM-negative. Class 1 means LNM-positive.**


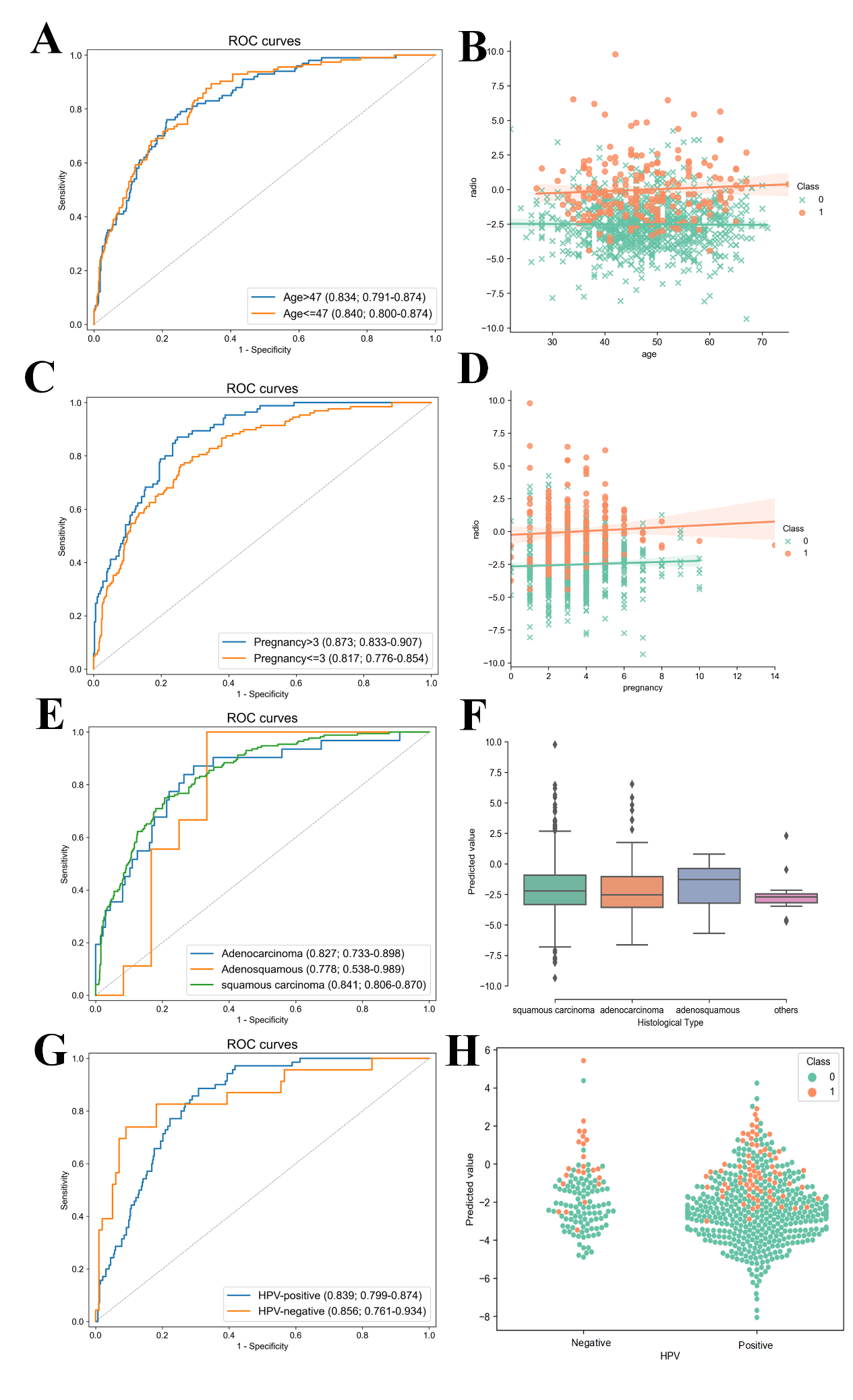


**Figure S6. Subgroup analysis on (A)different centers and (B) different CT manufacturers.**


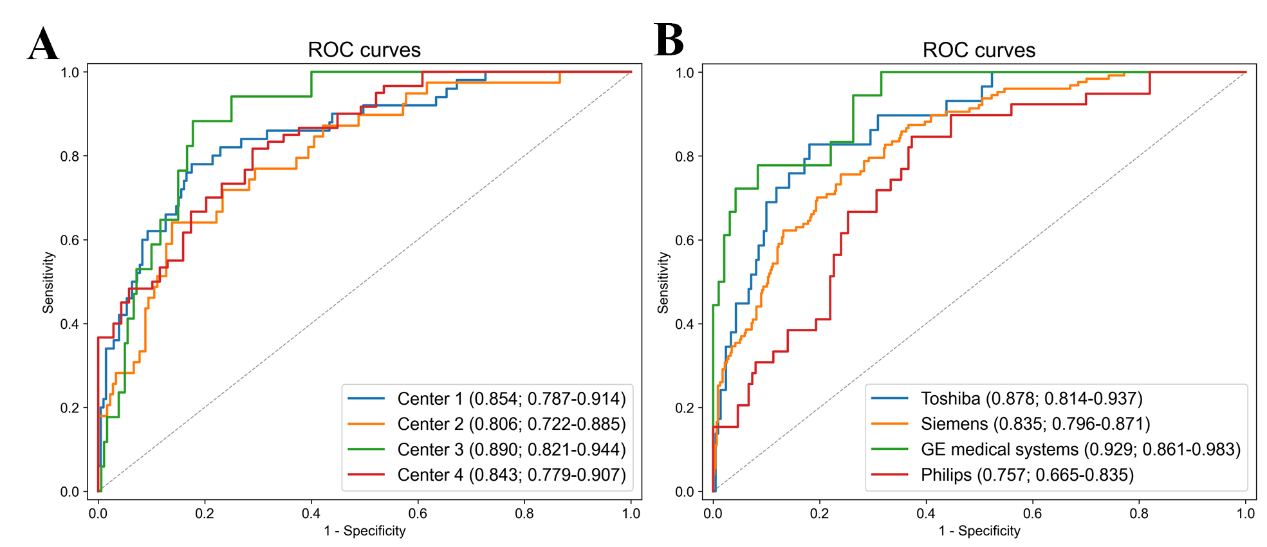


**Figure S7. Kaplan-Meier curve of overall survival for FIGO stage in follow-up cohort.**


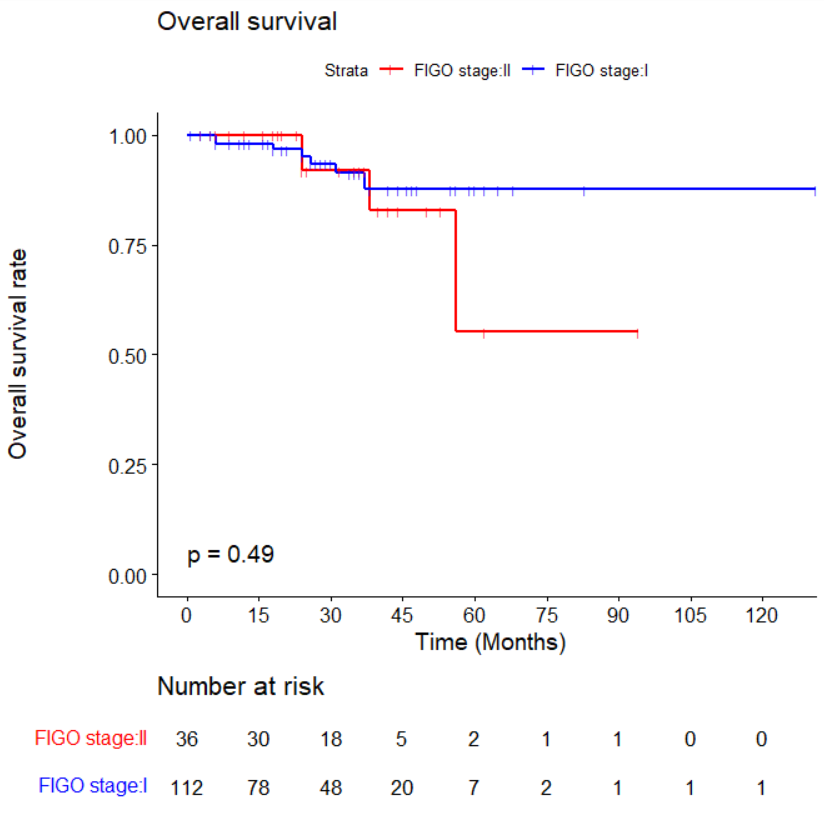

Supplement: Supplementary file 1 — Supplementary A1. The inclusion and exclusion criteria of this study Supplementary A2. The dataset partition and sample size estimation Supplementary A3. Region of interest segmentation and data preprocessing Supplementary A4. Evaluation of the models Supplementary A5. Training details of the three deep learning networks Supplementary A6. Handcrafted features extraction and Sig_radiomic building Supplementary A7. The prognostic analysis of DLN Table S1. Detailed information of the data in each center Table S2. Clinical characteristics in the training cohort, validation cohort and external testing cohorts Table S3. Performance of deep learning and radiomic signatures in all cohorts Table S4. The logistic linear regression of features in DLN Table S5. Performance of the DLN and the diagnoses of gynecologists in all cohorts. Figure S1. The Flowchart of this multicenter study. Figure S2. The ROC curves of different signatures in all cohorts. Figure S3. The performance of the constructed models in all cohorts. Figure S4. Venn diagram comparing the performance of DLN with the diagnoses of gynecologists. Figure S5. Subgroup analysis of clinical characteristics. Figure S6. Subgroup analysis on (A)different centers and (B) different CT manufacturers. Figure S7. Kaplan‐Meier curve of overall survival for FIGO stage in follow‐up cohort. [file CTM2-12-e938-s001.docx]
